# Crosstalk of ferroptosis and oxidative stress in infectious diseases

**DOI:** 10.3389/fmolb.2023.1315935

**Published:** 2023-12-07

**Authors:** Yibao Hu, Bisha He, Qian Cao, Yue Li, Yun Tang, Ting Cao, Binfeng Peng, Xiangping Zhou, Shuangquan Liu

**Affiliations:** Department of Clinical Laboratory Medicine, Institution of Microbiology and Infectious Diseases, The First Affiliated Hospital, Hengyang Medical School, University of South China, Hengyang, Hunan, China

**Keywords:** ferroptosis, oxidate stress, mechanism, infectious diseases, target therapy

## Abstract

Ferroptosis is a type of programmed cell death that pathogens can leverage to enhance their replication, transmission, and pathogenicity. Hosts typically combat pathogenic infections by utilizing oxidative stress as a defense mechanism. Nonetheless, some pathogens can trigger considerable oxidative stress while infecting, inducing an intense inflammatory response in the host’s immune system and activating cell death. The process of ferroptosis is closely linked to oxidative stress, with their interaction exerting a substantial impact on the outcome of infectious diseases. This article presents an overview of the interrelated mechanisms of both Ferroptosis and oxidative stress in infectious diseases, identifying potential targets for treating such diseases in the context of their interaction.

## 1 Introduction

**FIGURE 1:**
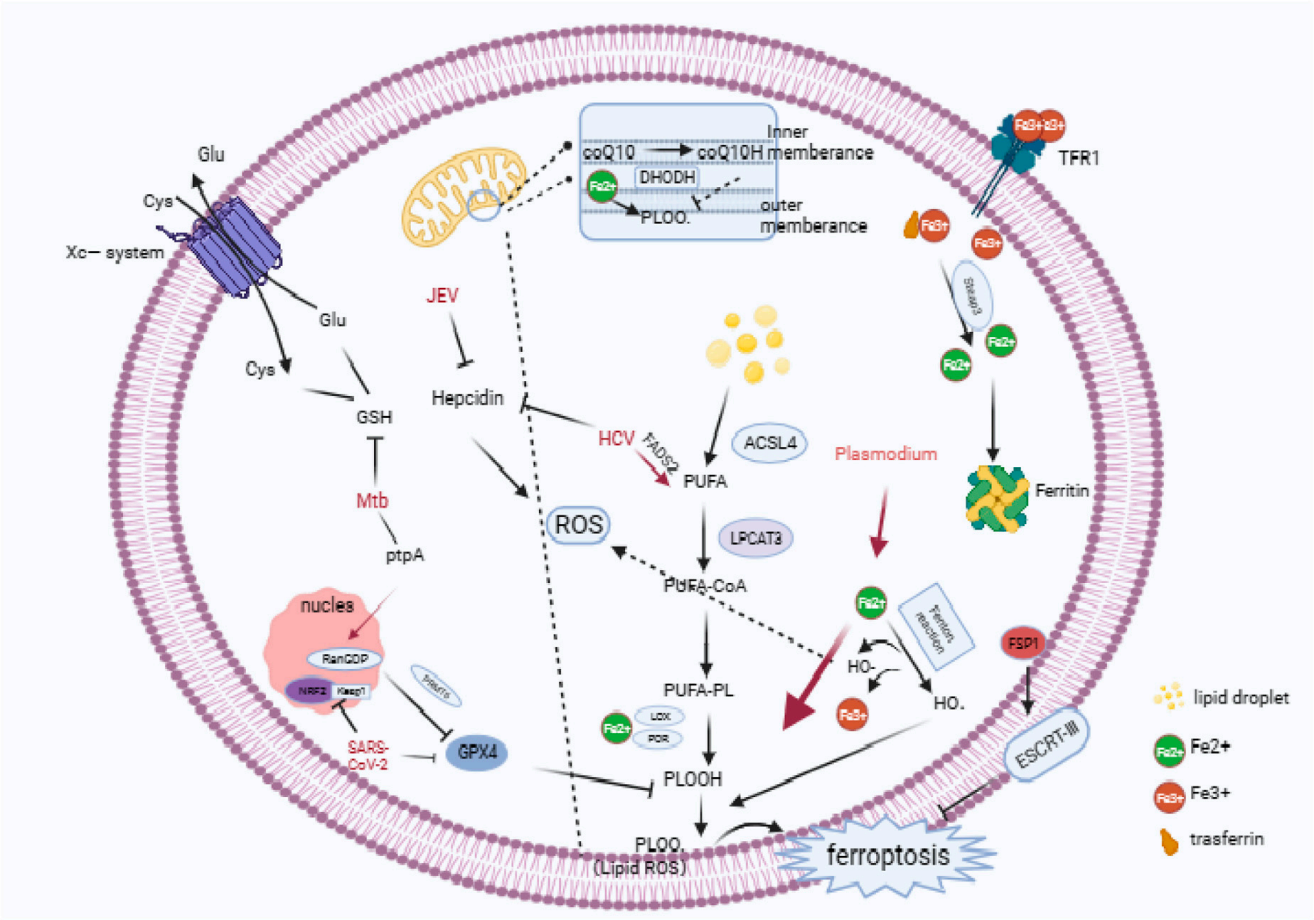
Cascade reactions in ferroptosis, the antioxidative systems involved in ferroptosis, and an overview of the pathogenic mechanisms employed by microorganisms in ferroptosis. (Ferroptosis is characterized by an abundance of ROS, and the overloading of intracellular iron is pivotal in the onset of ferroptosis. TfR1 mediates the entry of iron into the cell, which is then converted to ferrous iron by STEAP3 in the endosome. Typically, excess ferrous iron is sequestered in ferritin, but degradation of ferritin leads to an accumulation of ferrous iron. Ferrous iron, which is generated through the Fenton reaction, facilitates the production of LPO by HO·. This process elevates the levels of LPO, leading to ferroptosis. At the same time, iron ions catalyze phospholipids that consist of PUFA-PL through the Fenton reaction, resulting in PLOO·.In response to LPO, the cytoplasm contains three major antioxidant systems: GSH/Gpx4, ferroptosis suppressor protein 1/coenzyme Q10 (FSP1/CoQ10). Additionally, DHODH/CoQ10 and the GSH/Gpx4 system can exert antioxidant effects in mitochondria. In the SLC7A11-GSH-Gpx4 axis, cysteine is transported into cells via the XC-reverse transporter, and Gpx4 functions as a primary antioxidant enzyme during ferroptosis. Both SARS-CoV-2 and Mtb inhibit the expression of GSH or Gpx4, causing host cell ferroptosis to facilitate replication or spread. Plasmodium infection results in an overload of iron ions, leading to an increase in ROS production and the formation of LPO. HCV and JEV can promote the accumulation of iron ions in cells by down-regulating Hepcidn.) Technical abbreviations have been explained on first use, and language is clear, concise, objective, and grammatically correct. No biased or emotional language is employed, and the text adheres to conventional academic structure and formatting.

Ferroptosis exhibits three key characteristic features in its mechanism: the disruption of the Fe2+ steady state, the unrestricted generation of lipid peroxidation (LPO), and the inhibition of the antioxidant enzyme system. Disrupting the Fe2+ steady state is a crucial driver of Ferroptosis and a critical regulator of LPO and redox homeostasis. Enzymes responsible for phospholipid peroxidation, such as lipoxygenase (LOX) and cytochrome P450 reductase (POR), require iron for catalysis (Conrad and Pratt, 2019). A significant amount of Fe2+ can react with hydrogen peroxide in the Fenton reaction (Fe2+ + H2O2 → Fe3+ + -OH + OH-) to generate highly reactive hydroxyl radicals (-OH) (Cheng and Li, 2007). These radicals play an essential role in initiating lipid peroxidation. Phospholipids on cell membranes containing unsaturated fatty acids (PUFA-PLs) are susceptible to -OH and form PLOOHs accordingly. These PLOOHs can transfer oxidizing groups that compromise the integrity of the lipid on the cell membrane, leading to cell rupture and promoting Ferroptosis (Su et al., 2019; Stockwell, 2022). Furthermore, Ferroptosis utilizes a significant antioxidant enzyme system, which is mediated by glutathione peroxidase 4 (Gpx4). Gpx4 facilitates the transformation of phospholipid peroxides that contain hydroperoxide groups (PLOOH) into non-toxic PL alcohols (P-LOH). This aids in the removal of LPO and prevents the onset of Ferroptosis. Glutathione (GSH) acts as the main reducing substrate for GPX4 and is composed of cysteine, glutamate (Glu), and glycine. Intracellular cysteine functions as the precursor that limits the rate of transport by the Xc-system of the cell membrane (Li et al., 2022). Also, an enzyme within the mitochondrial membrane, DHODH, can reduce coenzyme Q10 to coenzyme Q10H2, which inhibits LPO (Mao et al., 2021). Oxidative stress results from an imbalance between the body’s production and elimination of reactive substances, such as hydrogen peroxide and oxygen free radicals (Sies, 2021). When the body’s antioxidant system is unable to counteract the effects of the oxidative system, cells experience oxidative stress. This leads to cellular organelle dysfunction and intracellular environment disruption (Lennicke and Cochemé, 2021). Reactive oxygen/nitrogen species (ROS/RNS), which are created by oxidative stress, can act as critical second messengers and participate in signal transduction pathways (Forman and Zhang, 2021). Although oxidative stress triggers the innate immune response, evidence suggests that increased levels of ROS may promote pathogen replication. Nonetheless, this is a delicate equilibrium, since excessive oxidative stress may harm host cells, facilitating the spread of pathogens. In contrast, hosts may exploit cellular damage resulting from oxidative stress to induce a killing effect on pathogens (Figure 1). In this review, we discuss host and pathogen effects under the influence of oxidative stress and Ferroptosis, and further elucidate the crosstalk between oxidative stress and Ferroptosis in the context of infection.

## 2 Ferroptosis and infectious diseases

### 2.1 Ferroptosis

Ferroptosis presents itself through mitochondrial shrinkage, reduced mitochondrial ribs, and rupture of the cell membrane (Yang and Stockwell, 2016). Host cells can become susceptible to ferroptosis due to disturbances in iron metabolism that inhibit iron efflux and/or increase iron uptake. During this process, Fe3+ is transported into the cell via transferrin receptor 1 (TfR1) and membrane transport protein 1. The export of Fe2+ to the extracellular space is managed by Ferroportin (FPN). The deterioration of FPN leads to the discharge of surplus iron ions into the cell, which, in turn, initiates the generation of LPO through the Fenton reaction. The formation of polyunsaturated fatty acid phospholipids (PUFA-PLs) is mandatory to induce LPO. Consequently, lipid metabolism is intimately associated with iron-dependent death. Two essential elements regulate the enzymatic synthesis of PUFA-PL: acyl coenzyme A synthetase long-chain family member 4 (ACSL4) and lysophosphatidylcholine acyltransferase 3 (LPCAT3) (Dixon et al., 2015; Doll et al., 2017). PUFA-PLs are at the forefront of LPO and deactivating ACSL4 or LPCAT3 can diminish the incidence of ferroptosis (Figure 1).

### 2.2 Role of ferroptosis in infectious diseases

#### 2.2.1 Tuberculosis (TB)

TB is an infectious disease caused by *Mycobacterium tuberculosis* (Mtb). The transmission of this disease occurs primarily through the air between individuals (He W. et al., 2017). In a study conducted by Amaral et al., they observed cell necrosis *in vitro*, in bone marrow-derived macrophages (BMDMs) from mice infected with a bacterial strain of *Mycobacterium tuberculosis* (H37Rv). This type of cellular demise differed from apoptosis and pyroptosis and was linked to elevated intracellular labile iron and lipid peroxide levels. Furthermore, there was a reduction in the expression of GSH and Gpx4, suggesting that MTB infection induced ferroptosis in BMDMs (Amaral et al., 2019). Further research showed that Mtb-infected mice with Gpx4 deficiency exhibited significantly increased lung necrosis and bacterial load. Conversely, overexpression of Gpx4 reduced bacterial load and tissue damage (Amaral et al., 2022). Furthermore, Li et al. identified a Mtb effector protein, protein tyrosine phosphatase A (PtpA), which induces ferroptosis to enhance Mtb pathogenicity and dissemination (Qiang et al., 2023). PtpA enters the host cell nucleus by interacting with host guanosine diphosphate (RanGDP) at its Cys11 site. Then, PtpA promotes the asymmetric dimethylation of histone H3 arginine 2 (H3R2me2a) by targeting protein arginine methyltransferase 6 (PRMT6), which inhibits the expression of GPX4 and induces ferroptosis (Qiang et al., 2023) (Figure 1). This demonstrates that Mtb can harm host immune cells via the ferroptosis pathway, therefore worsening the course of infection.

#### 2.2.2 Hepatitis C

Hepatitis C virus (HCV) is a significant factor in the development of chronic liver disease and liver cancer (Villanueva, 2019). The treatment for HCV usually involves the use of antiviral medications that specifically target the replicative enzyme components of HCV. The unique replicative enzyme of the HCV is regulated to a certain extent by LPO (Yamane et al., 2014). Yamane et al. discovered that LPO is a crucial and cell-permeable regulatory factor for HCV replication (Yamane et al., 2022). This regulated replication is propelled by the peroxidation of polyunsaturated fatty acids. Fatty acid desaturase 2 (FADS2) was determined to be the initial and rate-limiting enzyme in the synthesis of polyunsaturated fatty acids. These fatty acids can interact with iron and cause ferroptosis by enhancing high levels of endogenous LPO. Moreover, ferroptosis inhibitors like DFO or Fer-1 can decrease the binding affinity of antiviral drugs by suppressing LPO levels. Therefore, extensive LPO can significantly alter the conformation of HCV replication enzymes. This indicates that HCV replication can be inhibited by iron-induced oxidative stress. Furthermore, hepatic iron overload is common in HCV patients, which suggests that HCV infection may disrupt host iron metabolism homeostasis (Haque et al., 1996; Fujita et al., 2007; Kaito, 2007). A study proposed that increasing the presence of six-transmembrane prostate epithelial antigen 3 (STEAP3) can cause ferroptosis and mitigate HCV-infection-induced liver cirrhosis (Wang et al., 2019). STEAP3 is a metal reductase that converts Fe3+ to Fe2+. The mechanical characteristics of liver tissue not only affect its physiological processes, but also its progression in disease. Therefore, liver tissue mechanical factors have been studied as potential therapeutic targets and diagnostic markers (Wang et al., 2022). Shun et al. have pointed out that STEAP3 is a crucial detection target for tumor stromal mechanical heterogeneity and immune environment. Precisely, reducing matrix stiffness in HCC leads to STEAP3 downregulation and attenuation of ferroptosis that can enhance the anti-tumor effect mediated by PD-L2 (Wang et al., 2022). Although this study does not examine the direct impact of HCV, it implies that iron deficiency may hinder the onset and progression of HCV infection.

#### 2.2.3 Malaria

Malaria is an infectious disease caused by various species of Plasmodium parasites transmitted to mammals through infected mosquito bites. Upon infection, malaria parasites journey via the bloodstream to the liver, where they settle in liver cells, a phenomenon referred to as the hepatic phase of malaria (Lindner et al., 2012; Kaushansky and Kappe, 2015). Acquiring an abundance of iron in the liver is vital to the growth and development of malaria parasites (Drakesmith and Prentice, 2012). A study by Kain et al. (Kain et al., 2020) indicates that interfering with the SLC7A11-GPX4 pathway in liver cells infected with P. yoelii leads to ferroptosis induction. In contrast, NOX1 and TFR1 inhibition, the adverse path regulators lead to augmented infection during the hepatic phase. Therefore, SLC7a11-GPX4 disruption in the host selectively enhances LPO in infected cells, hence favoring malaria parasites clearance during the hepatic phase. Furthermore, LPO is significantly elevated in red blood cells infected with various types of malaria parasites. This condition comes with hemolysis and decreases in antioxidants such as GSH (Das and Nanda, 1999). In human patients with malaria, the level of iron-dependent LPO is higher in those infected with Plasmodium falciparum than those infected with Plasmodium vivax. The former is linked to malaria-induced anemia (Pamplona et al., 2007). In summary, the phenomenon of iron-mediated cell death plays a protective role in the battle against malaria during the hepatic phase of infection (Figure 1).

#### 2.2.4 Japanese encephalitis (JE)

Japanese Encephalitis Virus (JEV) is a flavivirus transmitted by mosquitoes and is the leading cause of viral encephalitis in Asia and the Western Pacific. Although the majority of JEV infections are asymptomatic, 20%–30% of clinical cases result in death while 30%–50% of survivors experience neurological, cognitive, or behavioral sequelae (Yun and Lee, 2014). Wang et al. reported that the ferroptosis pathway might be a factor in JEV infection of macrophages in their study (Wang et al., 2020). In a subsequent study, Singh and others found a significant increase in ferritin levels in cortical tissue samples from mice infected with JEV, along with iron accumulation in the cortex (Singh et al., 2023). Treatment with the iron chelator desferoxamine (DFO) resulted in reduced hepcidin levels and iron load in the mouse cortical tissue. Nevertheless, unlike infected animals, no changes in ferritin levels were noticed. The findings indicate that viral infections like JE lead to pathological processes involving iron ion overload and hepcidin regulation. Therefore, disturbances in host iron homeostasis are closely associated with JEV infection. However, additional research is necessary to investigate the molecular paths through which JEV leads to iron-induced cell death.

#### 2.2.5 COVID-19 pneumonia in novel coronavirus

COVID-19, triggered by Severe Acute Respiratory Syndrome Coronavirus 2 (SARS-CoV-2), has been linked to a marked expression of ferroptosis-related genes in lymphocytes, according to the findings of single-cell RNA sequencing (scRNA-seq) in COVID-19 patients (Huang et al., 2021). Furthermore, Liu et al. suggest that SARS-CoV-2 ORF3a encourages cellular vulnerability to ferroptosis via the Keap1-NRF2 axis, where the ORF3a protein plays a pivotal role in the pathogenesis of SARS-CoV-2 (Issa et al., 2020). Deleting ORF3a in animal models of SARS-CoV-2 infection reduces viral infection and lung tissue damage (Silvas et al., 2021). Studies indicate a vital association between thrombus formation in COVID-19 patients, ROS and ferroptosis. To decrease thrombus formation, lower iron ion levels or remove ROS production. One of the most frequent complications of COVID-19 is acute respiratory distress syndrome (ARDS), resulting in severe inflammatory responses and thromboembolism. COVID-19’s clotting process causes free iron in the blood to produce hydroxyl radicals, transforming fibrinogen into fibrin and facilitating clot development (Lipinski and Pretorius, 2013; Lodigiani et al., 2020; Oudkerk et al., 2020; Tal et al., 2020). These free iron ions also help to generate fibrinogen. The excessive accumulation of fibrinogen, akin to fibrin, may trigger arterial inflammation. Excessive iron ions contribute to increased production of ROS, along with pathological processes like thrombocytosis and elevated red blood cell viscosity. These factors all promote the formation of thrombi (Gill et al., 2019). Additionally, oxidized phospholipids (OxPLs) found in the lungs of patients with SARS-CoV-2 may contribute to thrombus formation by inducing the expression of tissue factor (TF) in monocytes (Owens et al., 2012; Merad and Martin, 2020). In summary, targeting ferroptosis can be a vital therapy for treating SARS-CoV-2 infection, providing new possibilities for managing COVID-19 clinically.

## 3 Oxidative stress and infectious diseases

### 3.1 Oxidative stress

Oxidative stress refers to the imbalance between oxidants and antioxidants that can cause protective or damaging effects on the oganism (Sies, 2017; Flohé, 2020). ROS play a significant role in the development of oxidative stress, and maintaining a dynamic balance between their production and clearance within cells is vital for cellular redox homeostasis (Lim and Leprivier, 2019). This article discusses the chemical entities present in the ROS spectrum, including oxygen (O2), superoxide anion radical (O2-), hydrogen peroxide (H2O2), and hydroxyl radical (•OH) (Valko et al., 2007; Rahal et al., 2014). ROS is an encompassing term that encompasses various oxidation molecules with divergent properties and biological functions, such as reactive nitrogen, sulfur, carbon, selenium, and halogen species (RHS). They participate in redox reactions and induce oxidative modifications on biomolecules, contributing to redox signaling and biological functions (Sies et al., 2022). The Electron Transport Chain (ETC) in mitochondria is a primary source of intracellular ROS. The, ETC., consists of multiple protein complexes I-IV, which are located within the mitochondria. During this process, a small percentage (1%–2%) of electrons are inevitably leaked from the, ETC., reducing surrounding O2 to superoxide ROS (O2-) (Chakrabarty and Chandel, 2021). In cells undergoing regular metabolism, H2O2 is kept at a stable and regulated level. It is generated simultaneously with O2- by mitochondrial NADH-dependent systems, mitochondrial external NADPH-dependent systems, and several oxidases. Under normal physiological circumstances, ROS generation is maintained at low levels due to reactions that involve Superoxide Dismutase (SOD1 and SOD3 - copper/zinc superoxide dismutase; intracellular and extracellular, and SOD2 - MnSOD; mitochondrial). These enzymes dismutate O2· to H2O2. Additionally, antioxidant enzyme systems such as catalase, peroxiredoxins, and the glutathione/glutathione peroxidase system also make vital contributions to upholding redox homeostasis (He L. et al., 2017; Lee et al., 2019; Miyazawa et al., 2019). However, heightened levels of ROS can upset the homeostasis of the body’s redox system, resulting in cellular demise and potentially tissue harm (Le Belle et al., 2011; Scherz-Shouval and Elazar, 2011; Amaral et al., 2019; Anthony et al., 2020).

### 3.2 Role of oxidative stress in infectious diseases

Oxidative stress serves a dual purpose in infectious diseases (Koo and Garg, 2019; Mullen et al., 2020) Host cells generate multiple signaling molecules in response to foreign pathogens during infection. The notable signaling molecule, ROS, can guard against pathogen contamination at low to moderate concentrations. ROS can bind directly to the side chains of amino acids, including arginine, cysteine, lysine, and proline, to affect the structure and function of proteins within the pathogen. This leads to biological inactivity. ROS can also cause direct damage to DNA and proteins in pathogenic organisms, resulting in the elimination of bacteria (Nyström, 2005; Traoré et al., 2009; Feeney and Schöneich, 2012; Van Acker and Coenye, 2017). Excessive production of ROS can cause LPO of polyunsaturated fatty acids on the microbial cell membrane, aiding in the elimination of foreign pathogens (Boylan et al., 2008). However, pathogens can also cause oxidative damage to biomolecules, including lipids, proteins, and DNA, by creating an excess of ROS. This can result in death of host cells and damage to tissues (van der Vliet and Janssen-Heininger, 2014). Many pieces of evidence suggest that viruses have evolved mechanisms to regulate and counteract levels of ROS and to mitigate ROS-mediated effects, thus maintaining a favorable cellular environment for viral replication. However, further exploration is necessary to fully understand the molecular mechanisms underlying this process.

## 4 Role of crosstalk between ferroptosis and ROS in infectious diseases

The role of ferroptosis varies in different infectious diseases. On one hand, pathogens can deploy ferroptosis to harm the host, while on the other hand, the host can eradicate pathogen infections via ferroptosis. Increasing evidence supports the notion that ferroptosis is a two-sided sword in infectious diseases (Hassannia et al., 2019; Chen et al., 2021b; a). Pathogens can trigger excessive ROS generation through two major pathways. Pathogens induce iron overload in host cells through their metabolism, resulting in the Fenton reaction and significant ROS production. Additionally, pathogens inhibit host antioxidant enzymes/systems like GPX4/Xc-. ROS accumulation and PUFA-PL synthesis further accelerate LPO. Mitochondrial metabolism has been shown to produce ROS and PUFA-PL that generate LPO without inhibition, resulting in ferroptosis (Liu et al., 2023). Here, we use *Mycobacterium tuberculosis* and HCV to suggest that reducing oxidative stress can prevent pathogen-induced host cell ferroptosis. We also emphasize that antioxidant therapy protects against ferroptosis damage caused by pathogens.

### 4.1 The heme oxygenase-1 (HO-1) target has the potential to treat *Mycobacterium tuberculosis* infection

In the early stages of MTB infection, tuberculosis lesions are composed mainly of macrophages and granulocytes (Palanisamy et al., 2011). In addition, in the later stages of tuberculosis, a large amount of heme is generated, leading to cytotoxicity and inducing oxidative stress in cells (Immenschuh et al., 2017; Yang et al., 2022). Analysis of iron distribution in human lung tissues indicates that iron homeostasis in various microanatomical sites is severely disrupted by MTB, resulting in Fe 2+ overload (Chinta et al., 2018; Phelan et al., 2018). One study proposed that Mtb may induce ferroptosis in lung macrophages through a potential mechanism involving ROS-HO-1 (Ma et al., 2022).

Heme oxygenase-1 (HO-1) is an important antioxidant that is highly expressed in the lung and activated by various stress signals, such as ROS and inflammatory mediators (Kassovska-Bratinova et al., 2009). HO-1 is the product of the HMOX1 gene (Kutty et al., 1994). The promoter region of the HMOX1 gene contains an antioxidant response element (ARE), which is critical for the expression of HO-1. It is composed of the BTB and CNC homolog 1 (Bach1) transcription factor and one of the three small musculoaponeurotic fibrosarcoma genes (MafF, MafG, or MafK). Under normal conditions, BACH1 forms a heterodimer with Maf, and Maf binds to the Maf recognition element (MARE) in the promoter region of the target gene to inhibit transcription (Zhang et al., 2018). In cases of abundant heme production, heme binds to the heme-binding region of BACH1, inducing dissociation of BACH1 from the heterodimeric inhibitory protein complex. Subsequently, BACH1 molecules translocate to the cytoplasm, undergo ubiquitination, and are subsequently degraded by the proteasome. This process allows Maf to form a homodimer with Nrf2 through the same ARE region, leading to transcriptional activation (Reichard et al., 2007). Activation of Nrf2 requires its partner Kelch-like ECH-associated protein 1 (Keap1). Under oxidative stress, Nrf2 is released from its cytoplasmic inhibitor Keap1 and translocates to the nucleus. Thus, the redox-dependent Keap1/Nrf2 system plays a central role in the induction of HO-1 in response to oxidative stress. HO-1 can degrade heme in cells, thereby exerting antioxidant effects and providing protection to cells. Heme is a complex of iron and protoporphyrin IX, and in aerobes it binds to hemoglobin, transporting oxygen molecules carried by hemoglobin and transferring electrons in the respiratory chain (Hamza and Dailey, 2012). In the lung, HO-1 can exert antioxidant effects to eliminate oxidative damage caused by *Mycobacterium tuberculosis* (Ma et al., 2022). HO-1 can metabolize excess protoporphyrin IX ring heme to biliverdin, carbon monoxide (CO) and Fe2+ (Gozzelino et al., 2010). In addition, HO-1 can upregulate iron-binding proteins, ferritin, and iron transport proteins to promote the efflux of unstable iron from cells, thereby reducing intracellular iron to mitigate oxidative stress (Paiva et al., 2012; Lanceta et al., 2013). Thus, targeting HO-1 may serve as a therapeutic strategy for tuberculosis.

### 4.2 The targeting of GSK3β/NRF2 may serve as a potential therapeutic target for the treatment of hepatitis C virus infection

HCV induces the activation of ROS, which leads to the activation of histone deacetylase (HDAC) activity. This leads to the deacetylation of transcription factor binding sites and histones. This state of reduced acetylation modulates hypoxia-inducible factors (HIFs), C/EBPα, and STAT3, thereby inhibiting hepcidin expression in HCV subgenomic replicon cells (Miura et al., 2008). Prolonged downregulation may contribute to increased iron absorption in the duodenum and promote iron accumulation in the liver during HCV infection (Sengupta and Seto, 2004). In addition, prolonged hepcidin deficiency disrupts the body’s iron homeostasis (Födinger and Sunder-Plassmann, 1999; Maggio et al., 2011; Jiang et al., 2015; Cheng et al., 2021; Huang et al., 2022). Hepcidin is involved in the pathogenic process of HCV. HCV replication leads to the accumulation of ROS, which exacerbates oxidative stress in infected liver cells. GSK3β plays a critical role in the regulation of oxidative stress. Jiag et al. reported on the GSK3β/Nrf2 signaling pathway in JFH-1 HCV-infected Huh-7.5.1 cells and liver tissue samples from HCV patients. The results suggest that HCV infection triggers an antioxidant response to Nrf2 (Jiang et al., 2015). The GSK3β-Nrf2 pathway may play a suppressive role in preventing ferroptosis by inhibiting oxidative stress (Huang et al., 2022).

## 5 Summary and outlook

Infectious diseases are a major challenge to human health. While current therapeutic approaches are numerous, they often fail to achieve the desired treatment outcomes. With the gradual exploration of oxidative stress and ferroptosis, new discoveries have opened new avenues for the treatment of infectious diseases. Factors such as GPX4 sites, glutathione and the Xc system have become new therapeutic entry points for infectious diseases. Especially in the occurrence of oxidative stress and ferroptosis, they share common factors and targets, providing new perspectives for the treatment of infectious diseases. From the study of external molecular compounds to the exploration of signaling pathways in oxidative stress and ferroptosis research, these efforts emphasize the importance of treating infectious diseases and provide scientific theoretical support for further research in this field. However, despite these efforts, the interplay between oxidative stress and ferroptosis requires further investigation. For example, understanding the specific signaling molecules through which toxic lipid peroxides ultimately lead to the onset of ferroptosis remains an area of research. More importantly, how can we regulate the extent of iron death to both help the host eradicate pathogens and minimize the physiological damage caused by ferroptosis? Therefore, a thorough exploration and study of the relationship between oxidative stress and ferroptosis is essential to uncover more viable strategies for the treatment of infectious diseases.
